# Personalized therapy in endometriosis — based on ERα or ERβ expression

**DOI:** 10.1186/s12916-024-03415-x

**Published:** 2024-05-30

**Authors:** Lei Zhan, Yunxia Cao

**Affiliations:** 1 Department of Obstetrics and Gynecology, The First Affiliated Hospital of Anhui Medical University, Hefei, Anhui China; 2 NHC Key Laboratory of Study On Abnormal Gametes and Reproductive Tract (Anhui Medical University), Hefei, Anhui China; 3 Key Laboratory of Population Health Across Life Cycle (Anhui Medical University), Ministry of Education of the People’s Republic of China, Hefei, Anhui China

**Keywords:** Endometriosis, Personalized therapy, Estrogen receptor α, Estrogen receptor β

## Background

Endometriosis, an Estrogen-dependent and progestogen-resistant chronic disease, is characterized by the transplantation and implantation of functional endometrial glands and stroma outside the uterus. This condition can cause chronic pelvic pain and infertility. Current treatment options for endometriosis include surgical removal of the lesions and estrogen suppression therapy. Surgery can offer temporary relief for patients experiencing severe chronic pelvic pain. Estrogen suppression treatment helps reduce symptoms and inhibits the growth of lesions in endometriosis patients [1]. Endometriosis lesions can be found throughout the peritoneal cavity and vary greatly in terms of size, color, appearance, location, and morphology. Despite this heterogeneity, researchers classify endometriosis lesions into superficial peritoneal (SUP), ovarian endometrioma (OMA), and deeply infiltrating endometriosis (DIE). The heterogeneity of endometriosis in gynecology results in some patients experiencing no response. Furthermore, these treatments often carry significant side effects [2]. Evaluating subtype-specific associated biomarker could be a promising approach to improve current treatment.

## Main text

In *BMC Medicine*, a recent study utilizing gene expression data from endometriosis lesions demonstrates that ovarian endometrioma (OMA) subtype of endometriosis displays the most significant response to estrogen suppression treatment by directly affecting *ESR2* [3]. To compare gene expression in endometrial samples, this study utilized genome-wide gene expression data from the University of Turku (Dataset A) and gene expression data from the Gene Expression Omnibus GSE141549, as well as data collected from European ancestry patients attending clinics at the Royal Women’s Hospital or Melbourne in-vitro fertilization in Melbourne (Dataset B). The authors aimed to address the discrepancy between Dataset A and B by considering the genes that are significantly regulated across the menstrual cycle. Through advanced bioinformatic analysis, the gene expression correlation between Dataset A and B was found to be significant, which provides confidence in the quality and consistency of the datasets.

The study further examines the analysis of gene expression profiles based on menstrual stage, lesion subtype, and hormonal treatment. The authors point out that hormonal treatment has a significant impact on gene expression in the endometrium. Additionally, the gene expression profiles remain consistent regardless of the menstrual stage; however, they are able to distinguish between different lesion subtypes, with OMA being significantly different from both SUP and DIE. Moreover, the gene expression profile is altered by estrogen suppression medication in OMA, but not in SUP or DIE. The analysis of target receptors for hormonal medication reveals differential expression of *ESR2* in OMA and significant variation in co-regulated genes of *ESR2* between medicated and non-medicated OMA samples. These pieces of evidence suggest that OMA subtype endometriosis is more responsive to exogenous hormonal treatment compared to SUP or DIE subtype. Consistent with these findings, the development of *ESR2* ligand agents with anti-inflammatory properties, such as chloroindazole, showed promising treatment options by preventing lesion establishment through inflammation suppression, inhibition of angiogenesis, and neurogenesis in a mouse model without affecting fertility [4]. Despite identifying a specific response to hormonal treatments by estrogen-suppressive agents in their recent study, Marla and colleagues acknowledge that personalized treatment for endometriosis patients is still far from being achieved. In their study, they did not differentiate the effects of different hormone treatments separately, which means that the systemic suppression of estrogen and accompanying side effects cannot be avoided in patients with OMA subtype endometriosis who undergo hormone treatment. Furthermore, Marla and co-workers have not performed preclinical or clinical studies to validate their recommendations.

Estrogen receptor (ER) plays a crucial role in the development of endometriosis, with two key receptor subtypes involved: estrogen receptor α (ERα) and estrogen receptor β (ERβ). These subtypes are encoded by different genes, *ESR1* and *ESR2*, respectively. In endometriosis, both ERα and ERβ show abnormal expression and regulation compared to normal or eutopic endometrium [5]. Furthermore, ERα and ERβ exhibit distinct patterns of tissue expression, localization, and ligand specificities in endometriosis. This can be partially explained by the fact that ERα is believed to be the primary mediator of estrogenic action in endometrial glands and stroma. During the secretory phase, the levels of ERα decrease in all endometrial cell components, while the levels of ERβ decrease only in endometrial epithelial cells [6]. The unique characteristics of ERα and ERβ in endometrial glands and stroma offer potential options for personalized therapy in endometriosis.

The idea of treating endometriosis as a single entity was widely accepted and showed the potential to improve the current inadequate treatment. In 2018, Brichant et al. conducted a study that provided logical evidence suggesting that the heterogeneity of ERα and progesterone receptor distribution in lesions of DIE in untreated women or during hormonal treatments could explain why solely using endocrine treatments cannot cure this condition [7]. Later in 2020, Pluchino and colleagues conducted a study which observed that progestin treatment resulted in a decrease in ERα expression. The failure of ERα suppression by progestins predicted the severity of pain and recurrence at 1 year in DIE [8]. Pluchino’s results partially explain the progesterone resistance in endometriosis. Consistently, Harada and colleagues have suggested that SR-16234, a selective ER modulator, has the potential to be utilized as a treatment for pain associated with endometriosis. This is due to its reported estrogen receptor ERα antagonistic activity and strong affinity, combined with a weak partial agonistic activity towards the ERβ receptor [9].

## Conclusions

Recent studies suggest a new and promising approach to achieving precise and personalized therapy for endometriosis, focusing on ERα and ERβ expression. It appears that targeting ERα may be more appropriate for patients with DIE subtype endometriosis or those experiencing pain associated with endometriosis [10], while selectively targeting ERβ may provide greater benefits for endometriosis patients with OMA lesions. However, further research is needed to establish causality and understand the underlying mechanism for this variation. Additionally, future preclinical studies are necessary to assess the effectiveness and safety of ERα or ERβ agents. Developing an animal model based on the endometriosis subtype would significantly aid in the exploration and development of personalized treatments for this heterogeneous disease. Despite this, we remain convinced that each step taken towards subtype classification brings us closer to achieving personalized medicine for endometriosis.

## Data Availability

Not applicable

## Abbreviations

DIE: Deeply infiltrating endometriosis
ER: Estrogen receptor
OMA: Ovarian endometrioma
SUP: Superficial peritoneal
